# Transcriptome analysis of carnation (*Dianthus caryophyllus* L.) based on next-generation sequencing technology

**DOI:** 10.1186/1471-2164-13-292

**Published:** 2012-07-02

**Authors:** Koji Tanase, Chikako Nishitani, Hideki Hirakawa, Sachiko Isobe, Satoshi Tabata, Akemi Ohmiya, Takashi Onozaki

**Affiliations:** 1 National Institute of Floricultural Sciences, National Agriculture and Food Research Organization, Fujimoto 2-1, Tsukuba, Ibaraki, 305-8519, Japan; 2 National Institute of Fruit Tree Science, National Agriculture and Food Research Organization, Fujimoto 2-1, Tsukuba, Ibaraki, 8605, Japan; 3 Kazusa DNA Research Institute, 2-6-7 Kazusa-kamatari, Kisarazu, Chiba, 292-0818, Japan

**Keywords:** Carnation, *Dianthus caryopyllus* L*.*, Next-generation sequencing technology, SSR, Transcriptome

## Abstract

**Background:**

Carnation (*Dianthus caryophyllus* L.), in the family Caryophyllaceae, can be found in a wide range of colors and is a model system for studies of flower senescence. In addition, it is one of the most important flowers in the global floriculture industry. However, few genomics resources, such as sequences and markers are available for carnation or other members of the Caryophyllaceae. To increase our understanding of the genetic control of important characters in carnation, we generated an expressed sequence tag (EST) database for a carnation cultivar important in horticulture by high-throughput sequencing using 454 pyrosequencing technology.

**Results:**

We constructed a normalized cDNA library and a 3’-UTR library of carnation, obtaining a total of 1,162,126 high-quality reads. These reads were assembled into 300,740 unigenes consisting of 37,844 contigs and 262,896 singlets. The contigs were searched against an *Arabidopsis* sequence database, and 61.8% (23,380) of them had at least one BLASTX hit. These contigs were also annotated with Gene Ontology (GO) and were found to cover a broad range of GO categories. Furthermore, we identified 17,362 potential simple sequence repeats (SSRs) in 14,291 of the unigenes. We focused on gene discovery in the areas of flower color and ethylene biosynthesis. Transcripts were identified for almost every gene involved in flower chlorophyll and carotenoid metabolism and in anthocyanin biosynthesis. Transcripts were also identified for every step in the ethylene biosynthesis pathway.

**Conclusions:**

We present the first large-scale sequence data set for carnation, generated using next-generation sequencing technology. The large EST database generated from these sequences is an informative resource for identifying genes involved in various biological processes in carnation and provides an EST resource for understanding the genetic diversity of this plant.

## Background

Carnation (*Dianthus caryophyllus* L.) is one of the most popular cut flowers, and hundreds of cultivars are grown around the world. *Dianthus* is a genus of about 300 species in the Caryophyllaceae family. Several species, including *Dianthus caryophyllusD*. *barbatusD*. *chinensisD*. *plumariusD*. *superbus*, and their hybrids are widely used as horticultural cultivars [1]. The many flower varieties of carnation are divided into three groups (standards, sprays, and pot carnations) based on plant form, flower size, and flower shape. Standards have a single large flower per stem, whereas sprays have a larger number of smaller flowers; both types are used for cut flowers [2]. Pot carnation is a dwarf with many small flowers that is used as a potted plant. Most carnation cultivars are diploid (2*n* = 2*x* = 30), although some species of *Dianthus* are tetraploid or hexaploid [3-6]. According to the Plant C-values Database (http://data.kew.org/cvalues/), the total genome size (C-value) in carnation is 613 Mb (1.23 pg/2 C), which is four times that of the model plant *Arabidopsis* (0.30 pg/2 C) [7]. The genome of carnation is very small compared with those of other ornamental flowers, such as *Antirrhinum majus* (1,568 Mb), *Chrysanthemum morifolium* (9,384 Mb), *Ipomoea nil* (*Pharbitis nil*) (1,127 Mb), *Lilium longiflorum* (34,496 Mb), *Petunia hybrida* (1,642 Mb), *Rosa hybrida* (1,127 Mb), and *Tulipa gesneriana* (26,093 Mb).

Carnation cultivars are developed to be highly heterozygous so as to avoid the effects of inbreeding depression [8]. Most commercially important cultivars are hybrids that are propagated vegetatively. Carnation cultivars have been bred for attractive characteristics such as flower color, flower size, fragrance, and flower longevity. Carnation cultivars have a wide range of colors, including red, yellow, white, green, and brown. In addition, some flowers show marginal variegation, flecks, or sectors [9]. Recently, transgenic carnations with blue or violet flowers have been developed by the introduction of a heterologous flavonoid 3’, 5’-hydroxylase gene [10-12].

The vase life of cut flowers is one of the most important ornamental traits, because it affects consumer satisfaction and repeat purchasing. Carnation is a typical ethylene-sensitive flower [13,14], and its flower life is normally short (about 7 days) if preservatives are not used [15]. In the ethylene biosynthesis pathway, the conversion of *S*-adenosylmethionine (AdoMet) to 1-aminocyclopropane-1-carboxylate (ACC) and of ACC to ethylene are catalyzed by ACC synthase (ACS) and ACC oxidase (ACO), respectively. Transgenic carnations containing an antisense *ACO* gene exhibited low ethylene production and delayed petal senescence [16]. When the *Arabidopsis etr1-1* gene, capable of conferring ethylene insensitivity, was introduced into carnation, the transgenic carnation plants had reduced ethylene sensitivity caused by suppression of *ACO* expression, which prolonged flower life [17]. On the other hand, by repeated selection for lines with longer vase life, Onozaki et al. [18] produced two carnation cultivars (named ‘Miracle Rouge’ and ‘Miracle Symphony’) with improved vase life in which expression of three ethylene biosynthesis genes (*DcACS1DcACS2*, and *DcACO1*) was suppressed in flowers of both cultivars, which resulted in extremely low levels of ethylene production [18,19].

Expressed sequence tag (EST) sequencing is essential for functional genomics studies: it has been used to identify novel genes from a broad range of organisms and to provide an indication of gene expression levels in specific tissues. Currently, there are more than 69 million ESTs in the database (dbEST) at NCBI. Since the development of high-throughput DNA sequencing technologies, analyses using next-generation sequencers have been performed in cereals, legumes, and fruits, and large amounts of EST data have been submitted to various DNA databases. These studies have revealed that high-throughput DNA sequencing is a cost-effective approach to analyzing the ESTs of both model plants and non-model plants. Surprisingly, in *Arabidopsis*, at least 60 transcripts which did not exist in previous EST collections were identified by next-generation sequencing [20]. Furthermore, large-scale EST collection facilitates the design of microarrays and the high-throughput identification of simple sequence repeats (SSRs) and single-nucleotide polymorphisms (SNPs).

To identify the genes related to flower quality and important agronomic traits such as disease resistance, extensive gene expression profiling would be extremely valuable, but only 669 carnation ESTs were available on the NCBI website (http://www.ncbi.nlm.nih.gov/) at the early June 2012. Other genomics resources, such as markers and genomic sequences have yet to be developed for carnation. To improve the DNA sequence information available for carnation, we performed large-scale transcriptome sequencing of carnation using a next-generation sequencer (a Roche 454 GS FLX) and obtained more than 300,000 transcripts. This work will make a significant contribution toward plant physiology, biotechnology, and molecular genetics studies in carnation.

## Results and discussion

### EST sequencing and assembly

To maximize the range of transcript diversity, we extracted and pooled RNA from vegetative tissues, flowers at various developmental stages, and ethylene-treated flowers of ‘Francesco’, a major standard-type carnation cultivar. Two libraries, a normalized cDNA library and a 3’-UTR library, were synthesized from the RNA pool, and GS FLX 454 pyrosequencing runs were performed on these libraries. We obtained data from a cDNA library that had been previously sequenced by conventional Sanger (dideoxy-based) sequencing to identify SSRs [21]. The cDNA library was synthesized from RNA of aerial part of carnation. A total of 1,435,398 reads were obtained, of which 17,988 reads (1.25%) were obtained from Sanger sequencing (Table 1). After Cleaning (removal of adaptor sequences, poly(A) tails, etc.) of these sequences, the 454 sequencing of the normalized cDNA library generated 1,078,260 reads with an average length of 284 bp; 90,891 reads (8%) were less than 100 bp or less (Table 2). The 454 sequencing of the 3’-UTR library generated 339,150 reads with an average length of 323 bp; of these, 30,785 (9%) were less than 100 bp or less. These sequences resulted in a total 1,162,126 high-quality reads (Table 1). After clustering and assembly, 899,230 sequences were incorporated into 37,844 contigs, leaving 262,896 singlets, for a total of 300,740 unique sequences (Table 1). The average length of the contigs was 605 bp (range 117–3,850 bp). The 300,740 sequences were first compared with the sequences in the non-redundant NCBI database by using BLASTN. Next, for Gene Ontology (GO) classification, the contigs were annotated by searching for sequence similarities using BLASTX against *Arabidopsis* genes (TAIR v.7.0; http://www.arabidopsis.org); 62% of the contigs (23,380 sequences) had at least one BLASTX hit. The percentage similarity between the carnation sequences and those of *Arabidopsis* was highly dependent on the length of the query sequence, as was previously seen in *Eucalyptus*[22]: longer sequences gave higher percent similarity. Contigs of carnation transcripts that were longer than 117 bp may be of good quality for similarity searches (data not shown). Recently, a number of large-scale EST data sets have been successfully constructed from non-model plants, including maize, chestnut, olive, the medicinal herb *Artemisia annua*, and *Cucurbita pepo*, by using high-throughput sequencing with the GS FLX 454 sequencer [23-26]. We compared the sequence length of these results and ours, and found no great differences; therefore, we judged our carnation transcripts data to be of sufficiently high quality for further investigation.

**Table 1:** Summary of carnation transcripts data

Total reads^a^	1435398
Total high-quality reads	1162126
Reads in contigs	899230
Total contigs	37844
Singlets	262896
Total unique sequences (contigs plus singlets)	300740

**Table 2:** Size distribution of 454 sequencing reads after removal of adaptor sequences

	Normalized cDNA library		3′-UTR library	
Read length	Number of reads	%	Number of reads	%
≤100 bp	90891	8.4	30785	9.1
101–250 bp	382543	35.5	89507	26.4
251–500 bp	557048	51.7	177269	52.3
501–750 bp	47743	4.4	41584	12.3
≥751 bp	35	0.0	5	0.0
Total	1078260	100	339150	100

### Functional annotation

**Figure 1:**
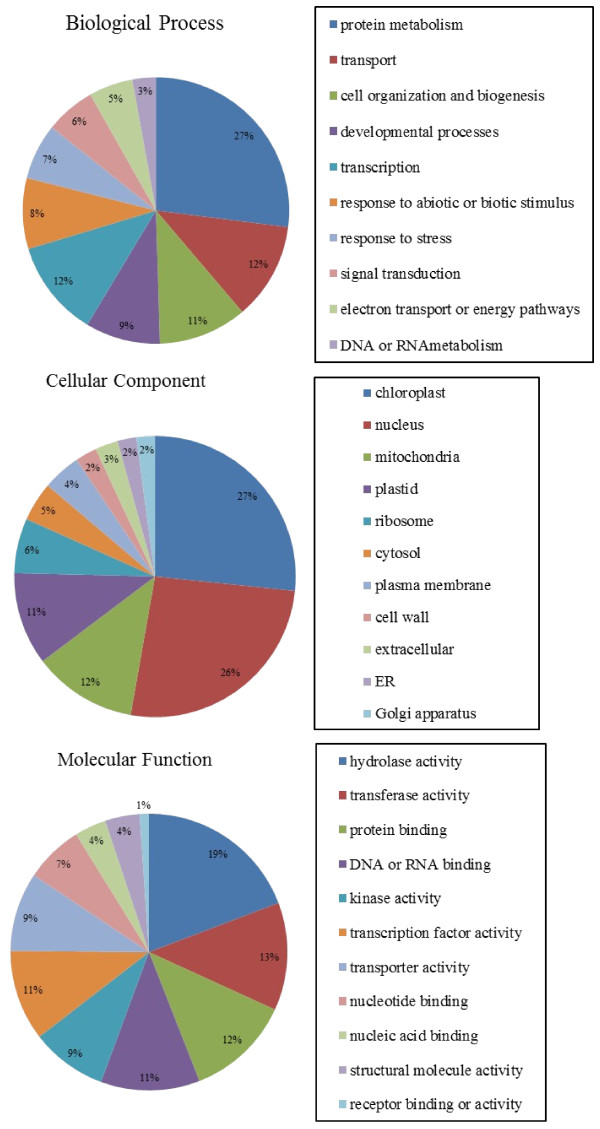
**Classification of transcripts into functional categories according to*Arabidopsis*Gene Ontology.A**: Biological Process, **B**: Cellular Component, **C**: Molecular Function. In the Biological Process category, “other biological processes”, “other metabolic processes”, and “other cellular processes” were omitted. In the Cellular Component category, “other cytoplasmic components”, “other cellular components”, “other membranes”, and “other intracellular components” were omitted. In the Molecular Function category, “other molecular functions”, “other binding”, and “other enzyme activity” were omitted.

GO has a controlled vocabulary that describes gene products in terms of their associated biological processes, cellular components, and molecular functions. We utilized the GO assignments of *Arabidopsis* gene models (the GO Slim classifications in TAIR) for assignment of putative functional roles to the 37,844 contigs of carnation. The top GO category matches for 17,584 genes of *Arabidopsis* were assigned to 23,380 (61.8%) of the contigs (E-value ≤ 10^-5^). These genes covered a broad range of GO categories (Figure 1), and some genes were assigned to more than one category. The most common assignments in the Biological Process category were protein metabolism (27%), transport (12%), transcription (12%), cell organization and biogenesis (11%), developmental processes (9%), and response to abiotic or biotic stimulus (8%). In the Cellular Component category, the largest classes were chloroplast (27%), nucleus (26%), mitochondria (12%), and plastid (11%). In the Molecular Function category, the most common assignments were hydrolase activity (19%), transferase activity (13%), protein binding (12%), DNA or RNA binding (11%), transcription factor activity (11%), and kinase activity (9%). The proportions of genes assigned to each GO category were very similar to those found in the genome annotation of *Arabidopsis*. The GO assignment analysis reinforces our assumption that a broad diversity of genes was sampled by using the selected tissues. Overall, these results of sequencing and functional annotation indicate that the large-scale sequencing technology is an efficient method for the transcriptome analysis of plants, especially those currently lacking other genomics tools.

### SSR marker discovery

SSRs are useful as random markers for population genetics research. To facilitate population genetics analysis and genetic mapping studies in carnation, we identified SSR motifs in our 454 data set. The reads from the 454 runs on the normalized cDNA library and the 3’-UTR library were used for EST-SSR discovery. The reads from conventional Sanger sequencing were not used for SSRs discovery in this study because SSR motifs from these reads were identified in a previous study [21]. Of the 1,417,410 sequences from 454 sequencing, 1,041,854 were incorporated into 109,951 contigs, leaving 375,556 singlets, for a total of 485,507 unique sequences after cleaning (removal of adaptors, poly(A) tail, etc), clustering, and assembly. A screen for the presence of SSRs was performed on this data set using the MISA program (http://pgrc.ipk-gatersleben.de/misa/). A search for di-, tri-, tetra-, and pentanucleotide repeats identified a total of 17,362 potential SSRs in 14,291 unigenes; that is, approximately 3% of the unigenes contained at least one of the SSR motifs included in the search (Table 3). This percentage was among the lower values seen for other species, in which approximately 3% to 20% of ESTs contained putative SSR motifs [23,27-29]. Finally, a total of 4,177 SSR primer pairs were designed from these unigenes using the MIRA3.2. program (http://mira-assembler.sourceforge.net/) (data not shown). Although further studies are needed to investigate and select for markers that show polymorphism, these data will provide powerful tools for the identification of markers linked to beneficial characters.

**Table 3:** Number of di-, tri-, tetra-, and pentanucleotide simple sequence repeats (SSRs) identified in 1,417,410 reads obtained by 454 sequencing

Dinucleotide repeat	Number of dinucleotide SSRs	%
AC/GT	150	15
AG/CT	321	33
AT/TA	170	17
CA/TG	81	8
GA/TC	250	26
Total	972	100
Trinucleotide repeat	Number of trinucleotide SSRs	%
AAG/CTT	616	6
AAT/ATT	652	7
AGA/TCT	787	8
ATC/GAT	614	6
CAA/TTG	811	8
GAA/TTC	871	9
TCA/TGA	768	8
Other trinucleotide repeats	4613	47
(<5% of each one)		
Total	9732	100
Tetranucleotide repeat	Number of tetranucleotide SSRs	%
AAAT/ATTT	176	7
AATC/GATT	165	7
ATTA/TAAT	145	6
ATTG/CAAT	121	5
TCAA/TTGA	287	12
Other tetranucleotide repeats	1498	63
(<5% of each one)		
Total	2392	100
Pentanucleotide repeat	Number of pentanucleotide SSRs	%
AAAAT/ATTTT	401	9
Other pentanucleotide repeats	3865	91
(<5% of each one)		
Total	4266	100

Very few genetic markers for horticulturally important characters in the major ornamentals, including carnation, have been identified [30]. To our knowledge, only a few studies have reported SSR marker development in carnation [21,31-33]. Smulders et al. [32,33] developed 8 SSR markers from the EMBL database and evaluated the genetic diversity in *Dianthus* species. These SSR markers were also used for constructing a genetic linkage map of carnation [34]. Kimura et al. [31] developed a set of 13 SSR markers and demonstrated their usefulness for genetic identification and hybridity confirmation of interspecific crosses in *Dianthus* species. Recently, a comprehensive set of 4,323 SSR primer pairs, representing 178 unique marker loci in 16 linkage groups, was developed and experimentally validated for carnation [21]; one of these loci was identified as a quantitative trait locus for carnation bacterial wilt resistance. In general, SSRs derived from ESTs are tightly linked with functional genes that may control useful characters. Furthermore, SSR markers can contribute to the construction of genetic linkage maps, genetic identification, and parentage analysis in *Dianthus* species.

### Transcripts related to flower color

Red and yellow petal colors in higher plants are generally produced by anthocyanins and carotenoids, respectively, but species belonging to the order Caryophyllales show unique pigment composition in their flowers. In most of the Caryophyllales, red and yellow petal colors are derived from betalains; most of them accumulate neither anthocyanin nor carotenoids in their flowers. Carnation is an exception in that it accumulates anthocyanins and can express red and pink colors. The yellow petal color of carnation cultivars is derived from chalcone, a yellow flavonoid, rather than from carotenoids. Although chlorophylls are generally absent from the flowers of most plants, some carnation cultivars accumulate chlorophylls in their petals and have a green flower phenotype. It will therefore be interesting to investigate the expression of genes involved in the metabolism of these pigments in members of the Caryophyllales. The carnation EST database will provide useful information for future studies at the molecular level.

### Carotenoid and chlorophyll metabolism

**Figure 2:**
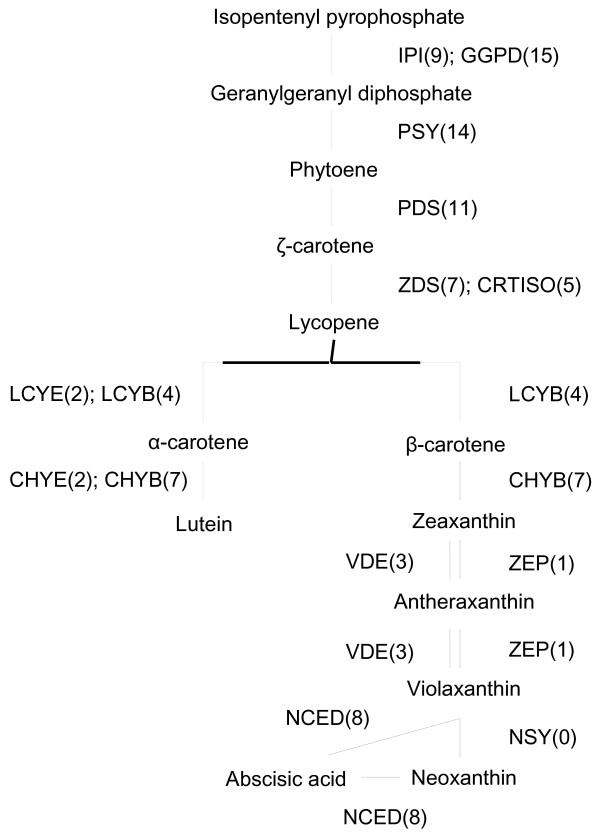
**Distribution of carnation transcripts in the carotenoid biosynthesis pathway.** Previously published sequences in GenBank belonging to the carotenoid biosynthesis pathway were used in BLAST searches to identify genes in the carnation EST database. Each enzyme name is followed in parentheses by the number of contigs homologous to gene families encoding this enzyme. IPI, isopentenyl pyrophosphate isomerase; GGDP, geranylgeranyl diphosphate synthase; PSY, phytoene synthase; PDS, phytoene desaturase; ZDS, ζ-carotene desaturase; LCYB, lycopene β-cyclase; LCYE, lycopene ϵ-cyclase; CHYB, β-ring hydroxylase; CHYE, ϵ-ring hydroxylase; ZEP, zeaxanthin epoxidase; VDE, violaxanthin de-epoxidase; CRTISO, carotenoid isomerase; NSY, neoxanthin synthase; NCED, 9-*cis*-epoxycarotenoid dioxygenase.

Carotenoids are isoprenoid compounds synthesized from isopentenyl diphosphate (IPP), a five-carbon isoprene unit. Because IPP is a precursor of various physiologically important compounds such as chlorophyll, tocopherol, gibberellin, and cytokinin [35], genes encoding isoprenoid biosynthesis enzymes might be expressed throughout the plant body. In *Arabidopsis*, all of the genes for isoprenoid biosynthesis are expressed in both flowers and leaves (TAIR: http://www.arabidopsis.org/). However, among the genes upstream of IPP, only *deoxyxyllulose 5-phosphate synthase* (*DXS*) was found in the carnation EST database. On the other hand, we found transcripts corresponding to most enzymes functioning downstream of IPP and leading to the synthesis of carotenoids. The database contained more than one transcript each for isopentenyl pyrophosphate isomerase, geranylgeranyl diphosphate synthase, phytoene synthase, phytoene desaturase, ζ-carotene desaturase, carotenoid isomerase, lycopene β-cyclase, lycopene ϵ-cyclase, β-ring hydroxylase, ϵ-ring hydroxylase, and violaxanthin de-epoxidase (Figure 2). Only one transcript was found for zeaxanthin epoxidase. Galpaz et al. [36] reported that multiple homologs of geranylgeranyl diphosphate synthase, phytoene synthase, ζ-carotene desaturase, and β-ring hydroxylase are present in tomato and expressed in a tissue-specific manner. It is of great interest to learn the tissue specificity of the multiple homologs of the carotenoid biosynthesis genes found in the carnation transcripts database.

Carotenoid catabolism produces diverse apocarotenoid compounds that are essential for plant growth and reproduction [37]. One category of these compounds (abscisic acid and strigolactone) is categorized as a plant hormone, and the others provide fruits and flowers with aromas and colors for attracting pollinators and seed dispersers. Such bioactive apocarotenoids are produced when carotenoids are cleaved by carotenoid cleavage dioxygenase (CCD). Analysis of the genome sequence of *Arabidopsis* led to the definition of nine clades of dioxygenases [38]. Five of these, the 9-*cis* epoxycarotenoid dioxygenases (NCEDs; NCED2, NCED3, NCED5, NCED6, and NCED9) are involved in the synthesis of the plant hormone abscisic acid. The remaining four are involved in the synthesis of the plant hormone strigolactone (CCD7 and CCD8), in aroma formation (CCD1), and in the regulation of carotenoid content in the flower (CCD4). The carnation transcripts database contained sequences corresponding to two types of NCEDs, which showed high sequence similarity to NCED2 and NCED5, and one type of CCD, which showed high sequence similarity to CCD1.

**Figure 3:**
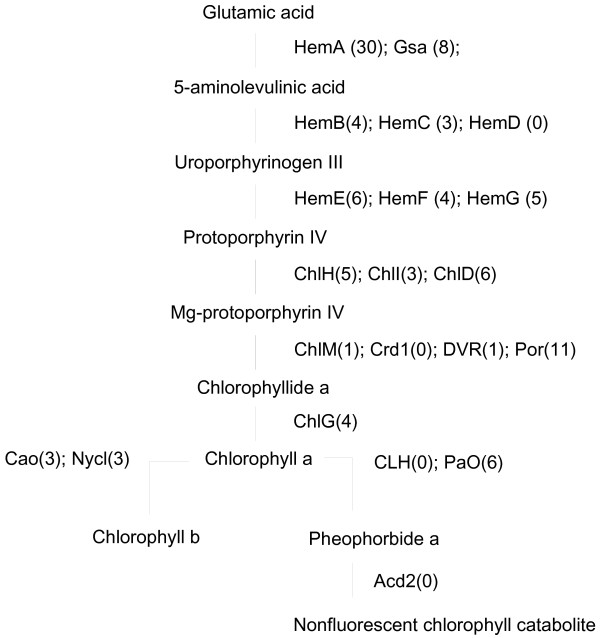
**Distribution of carnation transcripts in the chlorophyll biosynthesis and degradation pathways.** Previously published sequences in GenBank belonging to the pathways of chlorophyll biosynthesis and degradation were used in BLAST searches to identify genes in the carnation EST database. Names of enzymes (or subunits, in the case of Mg chelatase) are followed in parentheses by the number of contigs in different families of genes encoding these enzymes. HemA, glutamyl-tRNA reductase; Gsa, glutamate-1-semialdehyde 2,1-aminomutase; HemB, 5-aminolevulinate dehydratase; HemC, hydroxymethylbilane synthase; HemD, uroporphyrinogen III synthase; HemE, uroporphyrinogen III decarboxylase; HemF, coproporphyrinogen III oxidase; HemG, protoporphyrinogen IX oxidase; ChlH, Mg chelatase H subunit; ChlI, Mg chelatase I subunit; ChlD, Mg chelatase D subunit; ChlM, Mg-proto IX methyltransferase; Crd1, Mg-proto IX monomethylester cyclase; DVR, divinyl-protochlorophyllide reductase; Por, protochlorophyllide reductase; ChlG, chlorophyll synthase; Cao, chlorophyllide *a* oxygenase; Nycl, chlorophyll *b* reductase; CLH, chlorophyllase; PaO, pheophorbide *a* oxygenase; Acd2, RCCR reductase.

The chlorophyll metabolic pathway can be classified into three distinct phases: chlorophyll biosynthesis, the chlorophyll cycle, and chlorophyll degradation [39]. Numerous enzymes function in these processes, and most of the genes encoding these enzymes were represented in the carnation transcripts database. Among the 14 enzymes involved in chlorophyll biosynthesis, transcripts corresponding to 12 enzymes were found in the database; the only ones not represented were uroporphyrinogen III synthase and Mg-proto IX monomethylester cyclase (Figure 3). Transcripts for chlorophyllide *a* oxygenase and chlorophyll *b* reductase, both of which are involved in the chlorophyll cycle, were found in the carnation database. Among the enzymes involved in chlorophyll degradation, transcripts corresponding to pheophorbide *a* oxygenase were found, but chlorophyllase, pheophytinase, and red chlorophyll catabolite reductase (RCCR) were not.

Chlorophylls and carotenoids are essential pigments that play important roles in photosynthesis. In ‘Francesco’ carnation, high levels of these pigments were found in the leaves but not in the flowers (data not shown). Thus, the transcripts related to carotenoid and chlorophyll biosynthesis might have been derived from leaves. On the other hand, chlorophyll degradation is generally activated during leaf senescence. The absence of transcripts for some chlorophyll degrading enzymes might be explained by the fact that RNA was obtained from flowers and developing leaves but not from senescent leaves.

### Anthocyanin biosynthesis

**Figure 4:**
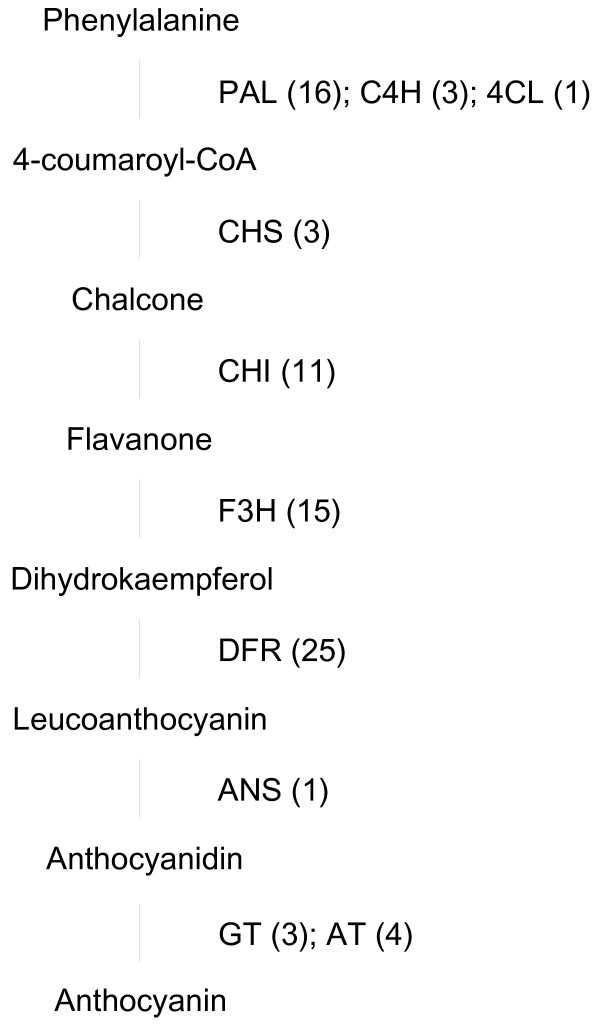
**Distribution of carnation transcripts in the anthocyanin (flavonoid) biosynthesis pathway.** Previously published sequences in GenBank belonging to the flavonoid biosynthesis pathway were used in BLAST searches to identify genes in the carnation EST database. Each enzyme name is followed in parentheses by the number of contigs homologous to gene families encoding this enzyme. PAL, phenylalanine ammonia-lyase; C4H, cinnamate 4-hydroxylase; 4CL, 4-coumaroyl:CoA ligase; CHS, chalcone synthase; CHI, chalcone isomerase; F3H, flavanone 3-hydroxylase; DFR, dihydroflavonol 4-reductase; ANS, anthocyanidin synthase; GT, anthocyanidin glucosyltransferase; AT, anthocyanin acyltransferase.

Anthocyanins are widely occurring colorants of fruits and flowers. Anthocyanidins, which are precursors of anthocyanins, are classified into six types: cyanidin, pelargonidin, peonidin, delphinidin, petunidin, and malvidin. Carnation flowers contain mainly pelargonidin- and cyanidin-type anthocyanidins [40-43]. Enzymes functioning in the anthocyanidin biosynthesis pathway have been well studied in many plants [12,44]. ESTs of all the enzymes involved in the anthocyanidin biosynthesis pathway (from phenylalanine to anthocyanidin) were present in the carnation transcripts database (Figure 4). Every enzyme in the pathway was represented by multiple transcripts except for 4-coumaroyl CoA ligase and anthocyanidin synthase, each of which was represented by a single EST.

Anthocyanidins are modified by glycosylation and acylation, to form anthocyanins. These modifications play important roles in changing flower color, increasing water solubility, and enhancing pigment stability. Recently, two types of glucosyltransferase have been identified and characterized in carnation [45,46]. Here, we found multiple transcripts encoding anthocyanidin glucosyltransferase and anthocyanin acyltransferase in the database. Thus, the carnation transcripts database will contribute to further investigations into the diversity of anthocyanin modification mechanisms.

### Betalain biosynthesis

Although the betalain biosynthesis pathway is poorly understood, several enzymes involved in this pathway have been identified and characterized [12,47]. Among them, an transcripts encoding dihydroxyphenylalanine (DOPA) dioxygenase was found in the transcripts database; further investigation will be necessary to verify if a part of the betalain biosynthesis pathway is active in the carnation flower.

### Ethylene biosynthesis and signaling

**Figure 5:**
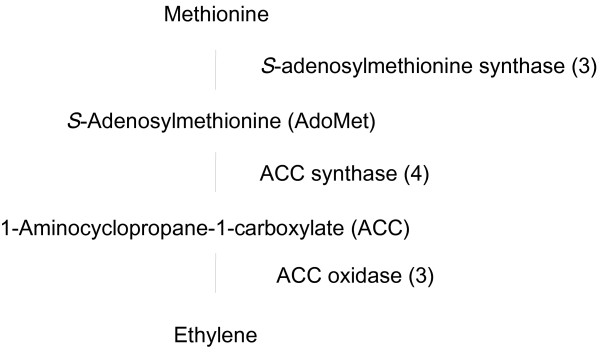
**Distribution of carnation transcripts in the ethylene biosynthesis pathway.** The initial precursor is the amino acid methionine, and key regulatory enzymes in the pathway are *S*-adenosylmethionine synthase, ACC synthase, and ACC oxidase. Previous published sequences in GenBank belonging to the ethylene biosynthesis pathway were used to identify genes in the carnation EST database by BLAST searches. Each enzyme name is followed in parentheses by the number of contigs homologous to gene families encoding this enzyme

Ethylene is a gaseous plant hormone with many important roles in growth and development [48], and is involved in flower senescence in many species [14]. The deterioration of the corolla in these species is accelerated by exogenous ethylene, and senescence is accompanied by an increase in endogenous ethylene biosynthesis [49]. The regulation of senescence in carnation, which is one of the most ethylene-sensitive flowers, has been investigated through the study of the expression of genes related to ethylene biosynthesis. In many plant species, including carnation, the pathway of ethylene biosynthesis is well characterized, having *S*-adenosylmethionine (AdoMet) as a starting compound and 1-aminocyclopropane-1-carboxylate (ACC) as an intermediate [50]. The conversion of methionine to AdoMet is catalyzed by *S*-adenosylmethionine synthase, the conversion of AdoMet to ACC by ACS, and the conversion of ACC to ethylene by ACO [51]. The carnation EST database contained multiple ESTs encoding each of these three ethylene biosynthesis enzymes (Figure 5). We cloned a cDNA encoding a novel *ACO* gene from the transcripts database constructed in this study (data not shown). Other carnation cDNA clones representing *S*-adenosylmethionine synthase, ACS, and ACO had high sequence similarity to those that have already been reported (Accession No. P24260, M66619, AF049138, AF049137, M62380). Additionally, we found ESTs corresponding to most ethylene signal pathway genes (Table 4). The database contained more than one EST each for ethylene receptors, *EIL*s (*ETHYLENE INSENSITIVE3-like*) and *ERF*s (*Ethylene-responsive-element–binding factor*). The members of these families are involved in the regulating various biological processes such as autocatalytic ethylene production, senescence, and various responses to stress through the ethylene perception [52]. Understanding the functions of these genes will help our understanding of regulation of ethylene dependent flower senescence in carnation.

**Table 4:** Distribution of carnation transcripts in the ethylene signaling pathway

Gene name	Number of contigs
Ethylene receptor	6
(*ETHYLENE INSENSITIVE3-like*) *EIL*	3
*Ethylene-responsive-element–binding factor* (*ERF*)	4

During flower senescence in carnation, a burst of ethylene production occurring in the gynoecium is followed by ethylene delivered to the petals, though the identity of the trigger signal molecule is still unknown. Autocatalytic ethylene production is induced by the signal, which in turn initiates downstream events in the senescence process such as lipid peroxidation and proteolytic activity [53,54]. Therefore, there is much interest in the regulation of senescence by the expression of genes related to ethylene biosynthesis. In many ethylene-sensitive flowers, ACS and ACO are key steps in ethylene production, and transcript levels of the corresponding genes are rapidly upregulated at the ethylene burst stage [49,50,54]. These findings suggest that *ACS* and *ACO* gene expression is transcriptionally regulated in carnation.

As mentioned in the Background section, the improved cultivars ‘Miracle Rouge’ and ‘Miracle Symphony’ have very long flower life (average 18 days) and show much lower ethylene production than normal cultivars [18]. In these improved cultivars, the expression levels of *DcACS1DcACS2* and *DcACO1* were low throughout the experimental period [19], but sequencing of genomic DNA did not detect any mutations in these genes (Tanase, unpublished). On the other hand, custom-made cDNA microarrays of carnation showed that some transcripts encoding transcription factors, including EIN3-like (EIL) transcription factors, a putative MYB-like protein, a zinc finger protein, a MYC-type protein, and MADS-box proteins, were upregulated during flower senescence [52]. In tomato, the MADS-box protein RIN (ripening inhibitor) regulates fruit ripening through direct activation of *LeACS2*[55,56]. Other transcription factors such as TOMATO AGAMOUS-LIKE 1 MADS-box protein and tomato HD-Zip homeobox protein, which regulate fruit ripening, probably control the expression of ethylene biosynthesis genes [57,58]. Our carnation database included many contigs related to transcription factor activity (11% of the Molecular Function ESTs) in the GO function analysis (Figure 1). Thus, the carnation transcripts database will contribute to further investigations into the regulation of ethylene biosynthesis and senescence programs in flowers.

## Conclusions

In this study, an EST database was developed to enable broad characterization of the carnation transcriptome. We detected 17,362 potential simple sequence repeats (SSRs) in 14,291 unigenes and identified transcripts corresponding to genes associated with carotenoid biosynthesis, chlorophyll biosynthesis and degradation, anthocyanin (flavonoid) biosynthesis, and ethylene biosynthesis and signaling. This collection of transcripts from carnation will be useful for the annotation of the forthcoming carnation genome sequence and provide a remarkable resource for genomics studies in Caryophyllaceae.

## Methods

### Plant materials and RNA extraction

Carnation (*Dianthus caryophyllus* L.) cultivar ‘Francesco’ was grown under natural daylight conditions in a greenhouse as described previously [15]. Each tissue was harvested from three plants. The following plant tissues were used: flower bud, flower (day 0 [full open flower], 3 days after full open, 8 days after full open, 4-h ethylene treated [10 μl·l^–1^, 20-h ethylene treated [10 μl·l^-1^), young and adult leaves, and stem (with shoot apex). Flowers contained sepals, petals, stamens and pistils. Tissues were immediately frozen in liquid nitrogen and stored at −80°C. Total RNA was extracted using the RNeasy Plant Mini Kit (Qiagen, Hilden, Germany). RNA concentration was estimated using an ND-1000 spectrophotometer (NanoDrop) (Thermo Scientific, Wilmington, DE, USA) and RNA integrity was evaluated using an Agilent 2100 Bioanalyzer (Agilent Technologies, Santa Clara, CA, USA).

### cDNA library construction for 454 sequencing

For 454 sequencing, we made a normalized cDNA library and a 3’ cDNA library in cooperation with Takara Bio (Otsu, Shiga, Japan). RNA isolated from each tissue was combined in equal proportions in a single pool in an attempt to maximize the diversity of transcriptional units sampled. The Clontech SMART system (Clontech, Mountain View, CA, USA) was used for cDNA synthesis from the total RNA.

To construct the normalized cDNA library, the cDNA was normalized by digestion with a duplex-specific nuclease. The normalized cDNA was amplified under the following conditions: 95°C for 20 s, followed by 25 cycles of 95°C for 5 s and 68°C for 8 min. The PCR primers were as follows: TD-5-P2 primer, 5’-GAGTGGCCATTACGGCCGGG-3’; biotinylated (T_18_)VN B-adaptor oligo, 5’-biotin-CCTATCCCCTGTGTGCCTTGGCAGTCTCAGTTTTTTTTTTTTTTTTTTVN-3’. After purification, the quantity of amplified cDNA was estimated using an ND-1000 spectrophotometer (NanoDrop) and the quality was evaluated using an Agilent 2100 Bioanalyzer. Approximately 5 μg of amplified cDNA was sheared into small fragments about 800 bp in length using an Acoustic Solubilizer (Covaris, Woburn, MA, USA). The cDNA library was constructed according to the manufacturer’s instructions in the Roche GS FLX Titanium General Library Preparation Method Manual.

For the 3’ cDNA library, we used the modified method of Eveland et al. (2008) [59]. Approximately 10 μg of amplified cDNA was sheared into small fragments about 800 bp in length with an Acoustic Solubilizer (Covaris). The cDNA fragments were selected by size, 400–1000 bp, using gel-cut and eluting them. The 3’ ends of the fragments were purified by using streptavidin-coated magnetic beads. Titanium A-adaptors (Roche, Basel, Switzerland) were ligated to the purified 3’ fragments, and the single-stranded 3’ cDNA was treated with 100 mM NaOH, neutralized, and purified. The quality of the 3’ cDNA library was assessed as described above for the normalized cDNA library. The 454 sequencing was performed according to the manufacturer’s instructions in the Roche GS FLX Titanium Sequencing Method Manual.

To construct the cDNA library for Sanger sequencing, poly(A)^+^ RNA from aerial part of carnation plant was purified using Oligotex-dT30 Super (Nippon Roche, Tokyo, Japan), and cDNA was synthesized by using a cDNA synthesis kit (Agilent Technologies) according to the manufacturer’s instructions. The size selection of cDNA and cloning into a pBluescript II SK^–^ plasmid were performed as previously described [60]. For generation of ESTs, plasmid DNAs were prepared from the colonies and sequenced using a BigDye Terminator Cycle Sequencing Ready Reaction Kit (Applied Biosystems, Foster City, CA, USA). The reaction mixtures were run on an automated DNA sequencer (ABI PRISM 3730; Applied Biosystems).

### Data analysis

Both the 454 sequences and Sanger sequences were trimmed of adaptor and low-quality sequence regions. All sequences were assembled and annotated by BLASTN searches of the NCBI database.

dditionally, the non-redundant set of consensus cDNA sequences represented by two or more reads (37,844 sequences) was annotated by BLAST searches of *Arabidopsis* cDNA databases. Functional classifications of these sequences were based on GO terms from the GO Slim classification in TAIR (http://www.arabidopsis.org).

### Accession numbers

Assembled transcripts of Carnation (*Dianthus caryophyllus* L.) were submitted to the Mass Submission System of DDBJ with the accession numbers FX296474 to FX334317.

### Detection of SSR markers

All of the assembled sequences from the 454 reads were used for detection of SSRs. SSRs in the total unique putative transcripts were detected by using the MISA program (http://pgrc.ipk-gatersleben.de/misa/), which accepts FASTA-formatted sequence files. Sequences containing di-, tri-, tetra-, and pentanucleotide repeats were selected.

## Competing interests

The authors declare that they have no competing interests.

## Authors’ contributions

KT prepared the cDNA libraries for 454 sequencing. SI and ST performed the conventional Sanger sequencing. HH, SI, and ST selected the SSRs and designed primer pairs. KT and CN performed the bioinformatics analysis. KT is the main coordinator of the carnation EST project and participated in the conception of the research together with AO and TO. KT was primarily responsible for drafting the manuscript. All authors read and approved the final manuscript.
